# Crystal structure of *Leishmania tarentolae *hypoxanthine-guanine phosphoribosyltransferase

**DOI:** 10.1186/1472-6807-7-59

**Published:** 2007-09-25

**Authors:** Paulo S Monzani, Stefano Trapani, Otavio H Thiemann, Glaucius Oliva

**Affiliations:** 1 Departamento de Física e Informática, Grupo de Cristalografia de Proteínas e Biologia Estrutural, Instituto de Física de São Carlos, USP, Caixa Postal 369, 13560-590, São Carlos – SP, Brazil; 2 Institut de Biologie Structurale *J.P. Ebel*, UMR 5075 CNRS, 41 rue Jules Horowitz, 38027 Grenoble cedex 1, France

## Abstract

**Background:**

Hypoxanthine-guanine phosphoribosyltransferase (HGPRT) (EC 2.4.2.8) is a central enzyme in the purine recycling pathway. Parasitic protozoa of the order *Kinetoplastida *cannot synthesize purines *de novo *and use the salvage pathway to synthesize purine bases, making this an attractive target for antiparasitic drug design.

**Results:**

The glycosomal HGPRT from *Leishmania tarentolae *in a catalytically active form purified and co-crystallized with a guanosine monophosphate (GMP) in the active site. The dimeric structure of HGPRT has been solved by molecular replacement and refined against data extending to 2.1 Å resolution. The structure reveals the contacts of the active site residues with GMP.

**Conclusion:**

Comparative analysis of the active sites of *Leishmania *and human HGPRT revealed subtle differences in the position of the ligand and its interaction with the active site residues, which could be responsible for the different reactivities of the enzymes to allopurinol reported in the literature. The solution and analysis of the structure of *Leishmania *HGPRT may contribute to further investigations leading to a full understanding of this important enzyme family in protozoan parasites.

## Background

Most known organisms synthesize purine bases by two pathways. The *de novo *biosynthesis pathway builds the purine nucleotide on 5-phosphoribosyl-alpha-1-pyrophosphate (PRPP). The *salvage *pathway recovers purines (adenine and guanine) from the degradation products of nucleotide metabolism and from hypoxanthine and xanthine. In contrast, parasitic protozoa such as the members of the *Kinetoplastida *order are auxotrophs for purine bases because the *de novo *biosynthetic pathway is completely absent [1]. They are therefore dependent on recycling pre-formed purine nucleotides and acquiring purines from the host. Central to the salvage pathway are the phosphoribosyltransferases (PRTases). In Kinetoplastids in general and *Leishmania *in particular, three PRTases are involved in the recycling of purine bases by the *salvage *pathway: hypoxanthine-guanine PRTase (HGPRT) (EC 2.4.2.8), adenine PRTase (APRT) (EC 2.4.2.7) and xanthine PRTase (XPRT) (EC 2.4.2.22) [2]. Several PRTases have been characterized from different organisms, but crystallization and structure determination have been accomplished for only two HGPRTs from Kinetoplastids, the parasite *Trypanosoma cruzi *[3] and *L. tarentolae *(present work).

PRTases are classified as Type I and Type II depending on their structural and catalytic features. The best-studied PRTases belong to the 'Type I' group, sharing a common α/β-fold at the PRPP binding motif and a flexible loop, besides a core region of at least five parallel β-strands surrounded by three or more helices [4,5]. The 'Type II' PRTases are composed of a mixed α/β N-terminal domain and an α/β barrel-like C-terminal domain. Currently, *Mycobacterium tuberculosis *and *Salmonella typhimurium *quinolinate PRTases are the only known structures in this group [6,7].

Considerable interest in the salvage pathway as a potential target for chemotherapy has been stimulated by the differences in purine base metabolism between mammalian hosts and protozoan parasites [2,8]. The creation of independent Δ*hgprt*, Δ*aprt *and Δ*xprt *null mutants by targeted gene replacement in *L. donovani *cells revealed that all three of the knockout strains generated are viable in the mouse macrophage model [9]. However, the Δ*hgprt*/Δ*xprt *double mutant *L. donovani *strain has less than 5% of the wild-type capacity to infect macrophages, establishing HGPRT and XPRT as essential for purine acquisition, parasite viability and infectivity in the mouse model [10].

*L. tarentolae *has been exploited as a model *Leishmania *for a variety of molecular, biochemical and evolutionary studies because of the ease of cell culture and genetic analysis of this species. In this paper we describe the three-dimensional structure of a *L. tarentolae *HGPRT protein and compare it with other HGPRT structures deposited in the Protein Data Bank. In view of the close phylogenetic relationship, the results will be of general significance as a model for other species of pathogenic *Leishmania*.

## Results and discussion

### General description

The refined crystallographic model of the dimeric HGPRT from *L. tarentolae *(PDB code – 1PZM) includes two protein monomers (chains A and B) in the asymmetric unit, with one molecule of GMP bound to the active site of each monomer. As summarized in Table 1, the overall quality of the model is good. The N-terminal regions (residues 1–19), the active site flexible loops (residues 95–107 from chain A and 94–105 from chain B), and the C-terminal regions (residues 202–210) containing a glycosome target sequence, are absent from the model, since they could not be located by inspection of the experimental electron density maps.

**Table 1:** Crystallographic data summary

***Crystal parameters***	
Space group	*P*2_1_2_1_2_1_
unit cell (Å)	*a *= 58.1, *b *= 85.4, *c *= 87.6
Matthews' volume (Å^3^/Da)	2.3
solvent content (%)	46.6
***Data reduction statistics***^(a)^	
high resolution limit (Å)	2.1
completeness (%)	94.9 (96.8)
*R*_*sym*_	0.093 (0.414)
<*I*/σ(*I*)>	15.6 (3.8)
observed unique reflections	24801 (1930)
redundancy	5.7 (5.5)
***Model refinement statistics***	
high resolution limit (Å)	2.1
*R*_*work*_	0.17
*R*_*free*_	0.21
number of protein atoms (2 monomers) ^(b)^	2715 (19)
number of solvent molecules	243
number of GMP atoms (2 molecules) ^(b)^	61 (13)
average isotropic *B*-factor (protein – Å^2^)	33.1
RMS deviations	
bond lengths (Å)	0.02
bond angles (°)	2.0
torsion angles (°)	16.8
improper angles (°)	0.1
Ramachandran plot (%)	
most favored	90.0
additionally allowed	8.7
generally allowed	1.3
disallowed	0
⟨real-space *CC*⟩ ^(c)^	0.94
σ_real-space *CC*_	0.07
No. Of residues with real-space *CC *< (⟨*CC*⟩ – σ_*CC*_)	37 (10.9%)
Directional atomic contact analysis ^(d)^	
all contacts *Z*-score	0.72
backbone-backbone contacts *Z*-score	-0.24
backbone-side chain contacts *Z*-score	0.02
side chain-backbone contacts *Z*-score	0.15
side chain-side chain contacts *Z*-score	2.03

^(a) ^Values between parentheses refer to the highest resolution shell (2.16--2.10 Å).

^(b) ^Values between parentheses refer to atoms with multiple occupancy counted once.

^(c) ^Correlation between (2*D|F*_*o*_|-*m|F*_*c*_|) and *F*_*c *_Fourier maps around protein residues only.

^(d) ^WHATIF second generation quality score.

### The monomer structure

**Figure 1:**
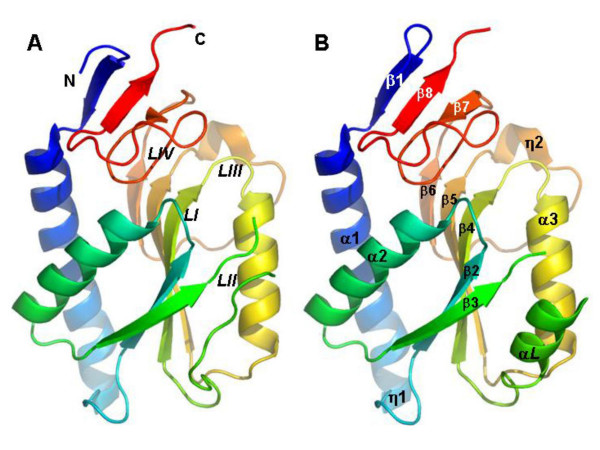
**Cartoon representation of *L. tarentolae *HGPRT tertiary structure**. The two monomers present in the crystal asymmetric unit are represented separately in the same orientation. Loops from the active site are labeled for monomer A and secondary structure elements are labeled for monomer B. Figures 1-6 were produced using PyMol [42].

*Leishmania *HGPRT is an α/β protein with the known PRTase type I fold. It is composed of two domains: a core domain containing the PRPP binding site [11-13], and a hood domain that binds the purine base substrate (Figure 1).

The core domain consists of a central five-stranded parallel β-sheet (strands β3, β2, β4, β5 and β6), with one α-helix packed on each side of the sheet (helices α2 and α3). A small 3_10 _– helix (η2) is present in the core domain. The central β-sheet is formed by two β/α/β motifs joined side-by-side through the first strand of each motif (β2 and β4). One further strand (β6) completes the central β-sheet. A phosphate binding site is present in the loops between the first β-strand and the α-helix of the β/α/β motif (called loops I and III respectively). Loops I and III are involved in binding the two terminal phosphates of PRPP [11-13]. *L. tarentolae *HGPRT Loop III residues Asp129, Ser130, Ala131 and Thr133 interact with the GMP phosphate group.

A flexible loop (loop II, residues 92–118), the function of which may be related to the formation of the transition state [11,14,15], comprises a region (residues 106–114) with good stereochemical and statistical values, which adopts different conformations in the two chains. In chain B, the polypeptide partially forms an α-helix (αL) similar to that in human HGPRT [4], while in chain A this helix is not observed and the polypeptide conformation resembles that found in *T. cruzi *HGPRT [3], *Tritrichomonas foetus *HGXPRT [16] and *E. coli *HPRT [17]. The different conformations adopted by these residues in the two monomers are consistent with the flexibility generally observed in the equivalent region (92–118) of other HGPRTases.

The hood domain contains both the N- and C-termini and is constituted by a small anti-parallel β-sheet with three strands (β1, β7 and β8). A loop (loop IV, residues 175–195) connects the β-strand β6 in the core domain to β8 in the hood-domain. This loop (IV) contains some of the residues that bind the base of GMP by hydrogen bonding with Val179 and Asp185 and by hydrophobic interaction with Phe178. The other connection between the core and the hood domain is made by a long α-helix (α1), which ends with a small 3_10 _– helix (η1). This helix appears to be important for the structural stability of HGPRT, since it interacts with all strands of the central β-sheet and with helix α2 of the core domain and is also involved in dimerization contacts.

### The dimeric interface

**Figure 2:**
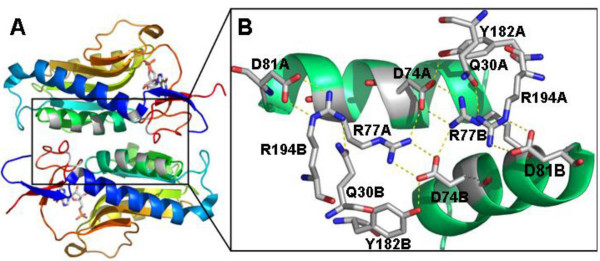
**Cartoon representation of the dimeric structure of *L. tarentolae *HGPRT**. (A) The GMP molecules in the active sites are also shown in dimeric structure. (B) Detailed representation (boxed region in A) of the saline bridges at the dimeric interface. The hydrogen bonds between Tyr182 and Asp74 and between Gln30 and Arg194 are also shown. Figure B is rotated relative to A for better visualization.

The dimerization interface of *Leishmania *HGPRT, shown in figure 2, is stabilized by a complex network of 45 non-bonded contacts and 12 hydrogen bonds involving 26 residues from dimer A and 25 from dimer B. Most of these residues are located in helix α2 and Loop IV. The 12 hydrogen bonds are formed between Lys66 (loop I) and Val88 (strand β3), Glu192 (loop IV) and Val86 (strand β3) and between residues Asp74 and Arg77 (helix α2), Asp81 (helix α2), Arg194 (loop IV), Asp74 and Glu30 (helix α1). Asp74 and Glu30 form intra-subunit H-bonds with Tyr182 and Arg194 (loop IV), respectively (Figure 2). We observed a similar network of interactions in the *T. cruzi *[3], *T. foetus *[16] and *E. coli *[17] homologues.

**Figure 3:**
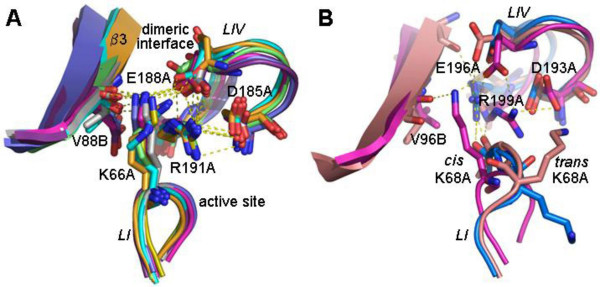
**Comparison of different HGPRT structures in the region surrounding the *cis *peptide bond**. (A) Superposed structures of HGPRT from *L. tarentolae *in gray (PDB 1PZM), *T. gondii *in purple (PDB 1QK5), human in magenta (PDB 1BZY), *T. cruzi *in green (DB 1TC2), *E. coli *in blue light (PDB), *P. falciparum *in blue dark (PDB 1CJB), *S. typhimurium *in orange (PDB 1J7J) and *T. tengcongensis *in yellow (PDB 1YFZ). The network of saline bridges is shown. (B) Comparison of different human HGPRT structures: the ImmGP-PPi-Mg complex in magenta (PDB 1BZY), which has a *cis *conformation, is superposed to the apoenzyme in blue (PDB 1Z7G) and the GMP complex in salmon (PDB 1HMP), which have a *trans *conformation.

Residues of the dimerization region are also involved in stabilizing the active site, particularly Arg191 (loop IV) and Lys66 (loop I) (Figure 3), suggesting a structural explanation for the fact that *Leishmania *HGPRT has been found exclusively as a dimer in both the presence and the absence of GMP [18]. The dimerization of HGPRT is an important step in the organization of loops I and IV. This is supported by mutants of the interface region in *Plasmodium falciparum *[19] and *T. cruzi *[20], which show drastically reduced catalytic activity.

Structural water molecules in each monomer (H_2_O1 and H_2_O18) stabilize the polar side chains of Thr37 and Tyr182 by hydrogen bonds that are found in a hydrophobic region formed by the Trp34, Val33, Phe71 and Phe78 side chains. Moreover, Thr37 and Tyr182 in both *Leishmania *and Human HGPRT form hydrogen bonds to neighboring Val33 and Asp74, respectively. This water molecule stabilization is exclusively observed in the *Leishmania *structure; in homologous structures, Thr37 is substituted by a hydrophobic residue.

### Comparison of HGPRT structures

The known HGPRT structures of *E. coli*, *S. typhimurium*, *Thermoanaerobacter tengcongensis*,* T. foetus*, *T. cruzi*, *P. falciparum*, *Toxoplasma gondii *and the human enzyme were compared with *L. tarentolae *HGPRT. The sequences were aligned (not shown) and the structures superposed.

A non-proline *cis *peptide bond between Leu65 and Lys66 from loop I is conserved in type I PRTases [3,5,11,16,17,21], where the amide nitrogen of Lys66 is exposed to the active site so that the peptide bond contributes two adjacent hydrogen bonds to the PRPP-metal complex [11]. However, our structural comparison of HGPRTs (Figure 3) suggests that the Lys66 *cis *conformation acts in the communication between monomers and drives the Arg191 side chain toward the active site into the correct position to bind PPi (Figure 3). Structures with a *cis *conformation in complex with PRPP as well as with PPi give strong evidence for this [11-14,22,23].

**Figure 4:**
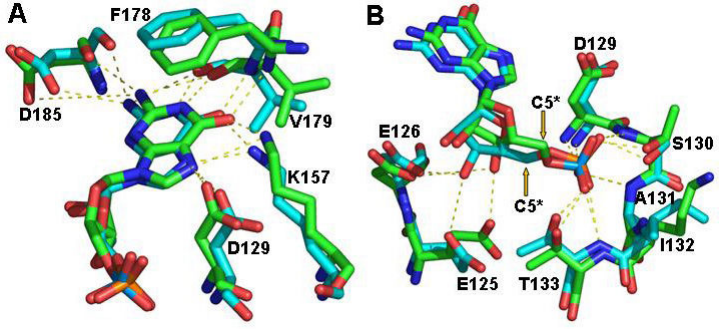
***L. tarentolae *HGPRT with bound GMP superposed on the human homologue**. H-bonds are shown as dotted lines. (A) Active site interactions of human (green) and *Leishmania *(blue) HGPRT with guanine. A purine base displacement is visible. (B) Interactions of phosphate group and ribose in the C3'-endo conformation. The arrows show differences in the arrangement of ribose carbon C5*.

Loop IV is conserved among the HGPRTs and interacts with the reaction product GMP through hydrogen bonds (Val179 and Asp185) as well as aromatic π-π stacking interactions (Phe178). An important contribution to the stabilization of GMP comes from Lys157 in strand β5, which makes two hydrogen bonds with the nitrogen base (atoms O6 and N7) (Figure 4). The two additional hydrogen bonds from Lys157 can be important in stabilizing the GMP in the active site if only three hydrogen bonds are formed between the nitrogen base and Loop IV. Moreover, Lys157 forms hydrogen bonds with Ala177 (Loop IV) allowing Loop IV to be properly positioned to interact with the base of GMP. Three other residues are conserved in the HGPRT structures: Gly181 and Asp185 are involved in Mg^2+ ^binding and Arg191 is involved in PRPP or PPi binding [11-14,22,23].

**Figure 5:**
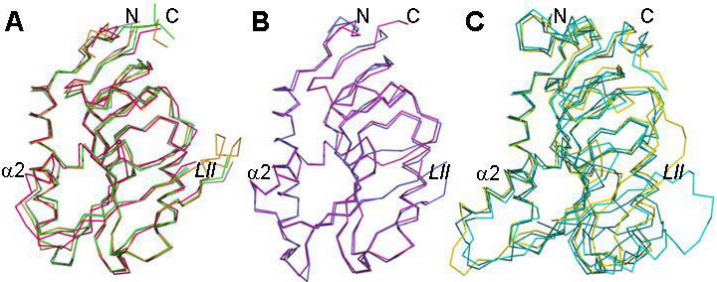
**Representation of three groups of superposed HGPRT structures in the same orientation**. Each group is characterized by a different size of the helix α2. (A) Group A includes structures of *S. typhimurium *(gray), *E. coli *(green), *T. tengcongensis *(orange) and *T. foetus *(magenta). This last organism presents an intermediate size of the α-helix between group A (prokaryotes) and group B (trypanosomatids). (B) Group B includes structures of *L. tarentolae *(pink) and *T. cruzi *(blue) presenting an α-helix intermediate in size between prokaryotes and human. (C) Group C includes structures of *P. falciparum *(yellow), *T. gondii *(green) and human (blue), showing the larger α-helix. The PDB used are the same as in Figure 3, with the exception of *T. foetus *(1HGX) and the human (1HMP) structures.

Three distinct HGPRT structural groups can be identified, mainly on the basis of the different sizes of helix α2 in the core domain and the C and N-terminus sequences of the hood domain (Figure 5): (I) the group comprising *S. typhimurium*, *E. coli*, *T. tengcongensis *and *T. foetus*, which have the shortest helices; (II) the trypanosomatids group (*Leishmania *and *T. cruzi*); and (III) the *P. falciparum*, *T. gondii *and human group, which have the longest helices.

The main differences between the *Leishmania *and human enzymes are found in the interactions between the GMP base and ribose and residues in the active site. Regarding the GMP base, we observe that in human HGPRT the N2 atom contacts both the oxygen and the carbonyl groups of Val187 and Asp193 (Loop IV), and the O6 atom forms a hydrogen bond with the NZ atom of Lys157. In contrast, the N2 atom of the GMP base in *Leishmania *HGPRT interacts preferentially with Asp193, and the Lys157 NZ atom interacts with both O6 and N7 of the base. The interaction distances are shown in Table 2. The Cα superposed in 11 residues interacting with GMP in the active site of *Leishmania *and human HGPRT result in an rmsd of 0.66Å. This analysis shows that residues from Loop IV have the largest rmsd and the guanine base shows a subtle orientation shift in this region between the two HGPRTs (Figure 4), particularly residues Asp129 and Asp185. The ribose of GMP in both the human and *Leishmania *enzymes is in the C3'-endo conformation used in the analysis of the bound GMP. In human HGPRT, the O3 atom of the ribose forms a hydrogen bond with OE2 of Glu133, while this hydrogen bond in *Leishmania *is formed with OD1 of Asp126. These differences are the result of the C5* atom arrangements (Figure 4), modifying the ribose position in the active site. According to our comparisons, the *Leishmania *HGPRT ribose is better stabilized by those interactions than the human ribose (Figure 4 and Table 2).

**Table 2:** Interaction distances between active site residues and GMP

**GMP Atom **	**Protein Atom **	**Residue hHGPRT^b^**	**Distance (Å)**	**Protein Atom **	**Residue *L*HGPRT^c^**	**Distance (Å)**
**Phosphate**						
O1A(O3P)^a^	OG1	Thr138B	2.7	OG	Ser130B	2.6
	N	Thr138B	3.1	N	Ser130B	2.9
O2A(O1P)^a^	N	Gly139B	2.7	N	Ala131B	2.9
	N	Asp137B	3.0	N	Asp129B	2.9
O3A(O2P)^a^	N	Thr141B	3.3	N	Thr133B	3.0
	OG1	Thr141B	2.9	OG1	Thr133B	2.6
	N	Lys140B	3.8	N	Ile132B	3.5
**Ribose**						
O3*	OE1	Glu133B	2.9	OE1	Glu125B	3.7
	OD1	Asp134B	4.7	OD1	Asp126B	2.8
**Purine**						
O6	NZ	Lys165B	2.3	NZ	Lys157B	2.9
	N	Val187B	3.1	N	Val179B	2.8
N1	O	Val187B	2.8	O	Val179B	2.8
N2	O	Val187B	3.3	O	Val179B	3.4
	O	Asp193B	3.2	O	Asp185B	2.8
	OD1	Asp193B	5.8	OD1	Asp185B	3.4
N7	OD2	Asp137B	3.6	OD2	Asp129B	4.4
	NZ	Lys165B	3.4	NZ	Lys157B	2.9

^a ^corresponding atoms in *Leishmania *HGPRT

^b ^Human HGPRT

^c ^*Leishmania tarentolae *HGPRT

### Leishmania HGPRT inhibition tests

Purine and pyrimidine analogs obtained from commercial sources were used to test the *Leishmania *HGPRT for possible inhibitors. All compounds tested resulted in high IC_50 _values, 8-aminoguanosine showing the best result (Table 3). One compound in particular, the antibiotic cefatoxime, has an IC_50 _value similar to allopurinol (Table 3).

**Table 3:** IC_50 _values of purine and pyrimidine analogs for *Leishmania *HGPRT

**Inhibitor**	**IC_50 _(μM)**
8-aminoguanosine	94
cefotaxime	180
allopurinol	194
azaadenine	210
formicin B	260
caffeine	336
5-bromodeoxiuridine	350
5-metilcitosine	412
Orotic acid	432
tubercidine	533
6-cloroguanine	838

HGPRT is a known activator of purine base analogs such as 6-mercaptopurine and allopurinol, and has been proposed as a target for antiparasitic chemotherapy [2,8]. Allopurinol is metabolized by HGPRT and incorporated into RNA during transcription, resulting in its degradation and inhibition of protein synthesis [24]. Allopurinol is metabolized more efficiently by the parasite HGPRT than the human homologue [25-27], prompting its evaluation in the treatment of leishmaniasis [28] and Chagas disease [25,29] with promising results.

**Figure 6:**
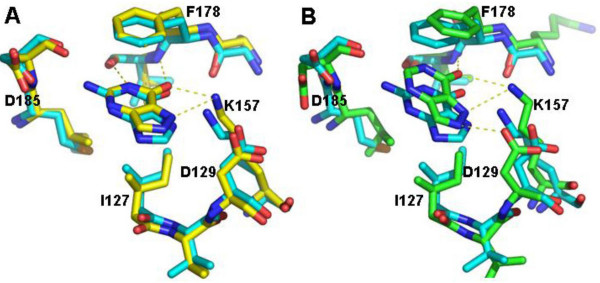
**Active site superposition**. (A) Active site superposition of HPP-bound *T. cruzi *(1TC2) (yellow) with GMP-bound *Leishmania *HGPRTs. Only the guanine from GMP is shown. (B) Active site superposition of HPP-bound human (1D6N) (green) with *Leishmania *(blue) HGPRT as in A.

The purine and pyrimidine analogs tested against *Leishmania *HGPRT present values in the high micromolar range (94–838 μM, Table 3). Allopurinol in particular has an IC_50 _of approximately 0.2 mM and is a more potent inhibitor of *Leishmania *than of *T. cruzi *HGPRT [11,13]. In the analysis of *T. cruzi *and human HGPRT with HPP (7-hydroxy-pyrazolo [4,3-D]pyrimidine, a compound similar to allopurinol) bound to the active site [11,13], subtle differences were observed in the binding that may be significant for novel compound design [13]. The superposition of HPP-bound active sites of *T. cruzi *and human HGPRT with GMP-bound *Leishmania *HGPRT showed that the guanine in the *Leishmania *structure is located in the same position as HPP in the homologous structures, with subtle differences that are more pronounced when the human and *Leishmania *homologues are compared (Figure 6). The differences between human and *Leishmania *HGPRT, like those in the GMP binding residues, as well as the GMP orientation and H-bond patterns in the active site, suggest that potential inhibitors may affect the protozoan enzyme differently from human HGPRT. This observation is supported by the high efficiency of incorporation of allopurinol by the *Leishmania *and *Trypanosoma *enzymes compared to human HGPRT.

## Conclusion

The X-ray structure of *L. tarentolae *HGPRT with GMP bound at the active site provides the template for comparison with the human enzyme. The subtle differences observed between the parasite and the human enzyme in the contacts with ligand can be explored for the design of potential parasite-specific inhibitors. The dimeric structure of *L. tarentolae *HGPRT shows an intricate hydrogen bond network important for enzyme stability and required for its activity. This analysis, together with the inhibition experiments using purine and pyrimidine analogs, has revealed differences in the binding efficiency of the enzyme active site that could be explored in the development of further inhibitors.

## Methods

### Protein expression, purification and crystallization

The recombinant HGPRT of *L. tarentolae *was over-expressed in *E. coli *BL21(DE3), and purified and co-crystallized with GMP in 19% PEG 4000, 20.6% isopropanol, 5% glycerol, 95 mM tri-sodium citrate, pH 5.6, as described previously [18]. Crystals grown under those conditions belonged to the primitive orthorhombic space group *P*2_1_2_1_2_1 _(*a *= 58.1Å, *b *= 85.4Å, *c *= 87.6Å, α = β = γ = 90°). Crystals of the free enzyme, which were also obtained at 18°C in 15% PEG 6000, 100 mM citrate, pH 5.1, diffracted poorly, and the use of additives led to crystals that diffracted only up to 3.0Å. Co-crystallization with GMP led to better-diffracting crystals up to a resolution of 2.1Å and these were used to solve the atomic structure of *Leishmania *HGPRT.

### Data collection and processing

*L. tarentolae *HGPRT crystals were transferred to a cryoprotectant solution without GMP, obtained by diluting the crystallization reservoir solution with 15% ethylene glycol (final concentration), mounted on nylon loops, and flash-cooled to 100 K. Diffraction data were collected at the Brazilian Synchrotron Light Laboratory with monochromatic X-rays (λ = 1.537Å) and a MAR345 image plate as detector [18]. Two sets of consecutive diffraction images (75 and 62 images respectively, with 1° rotation per image) from the same single crystal were collected and processed. The diffraction images were indexed and integrated using DENZO [30]. SCALEPACK [30] was used to scale and merge the data up to 2.1Å resolution. The data reduction statistics are summarized in Table 1.

### Structure solution and refinement

The crystal structure was solved by molecular replacement using the deposited structure of the dimeric HPRT of *T. cruzi *[3] as search probe (PDB entry 1TC1; 55% sequence identity). X-ray data in the 20–2.3Å resolution range were used and one dimeric probe was positioned in the asymmetric unit during the molecular replacement procedure (program AMoRe) [31]. The molecular replacement solution had a correlation coefficient of 57% and an *R*-factor of 43.5%. The molecular replacement model was refined iteratively in reciprocal and in real space using automated procedures and visual manipulation. Reciprocal space refinement was initially performed using the torsional simulated annealing procedure implemented in the CNS program [32] and continued using REFMAC5 [33] from the CCP4 suite (Collaborative Computational Project, Number 4, 1994), using a maximum-likelihood target with stereochemical restraints, two TLS [34] sets of parameters (one for each protein monomer in the asymmetric unit), and individually restrained isotropic B-factors. A set of structure factors representing 5% of the total experimental data was excluded from the reciprocal-space refinement target for purposes of cross-validation. Twofold non-crystallographic symmetry restraints were used initially and gradually removed during the refinement. The program O [35] was used to inspect the (*D*|*F*_*o*_|-*m*|*F*_*c*_|) and (2*D*|*F*_*o*_|-*m*|*F*_*c*_|) difference Fourier maps and to manipulate the model. Water molecules were added automatically to the model on the basis of the difference Fourier maps and distance criteria using the program ARP/wARP version 5.0 [36] from the CCP4 suite.

The stereochemical quality of the crystallographic model was constantly monitored during refinement using the PROCHECK [37], WHAT IF [38] and O [35] programs. The model/experimental map correlation was calculated using the MAPMAN^© ^program [39]. The refined TLS parameters and the residual isotropic atomic *B*-values were converted to atomic anisotropic displacement parameters using the program TLSANL [40] from the CCP4 suite.

### Inhibition tests

The HGPRT enzyme inhibition assay was performed for 1 min in a 1 ml reaction volume containing 100 mM Tris-HCl, 5 mM MgSO_4_, 1 mM PRPP, 0.04 mM guanine at pH 7.4 [41]. An extinction coefficient of 4.2 was used. Purine and pyrimidine analogues were tested using six inhibitor concentrations, in triplicate, to obtain the inhibition curve and calculate the IC_50 _values shown in Table 3.

## Abbreviations

APRT, adenine phosphoribosyltransferase; 

GMP, guanosine monophosphate; 

HGPRT, Hypoxanthine-guanine phosphoribosyltransferase; 

HPP, 7-hydroxy-pyrazolo [4,3-D]pyrimidine; 

PEG, poliethileneglycol; 

PPi, pyrophosphate; 

PRPP, 5-phosphoribosyl-alpha-1-pyrophosphate; 

XPRT, xanthine phosphoribosyltransferase; 

PRTase, phosphoribosyltransferase.

## Competing interests

The author(s) declares that there are no competing interests.

## Authors' contributions

OHT made the HGPRT expression construct and together with GO was project coordinator. PSM collected the expression, purification, crystallization and X-ray diffraction data. ST and PSM solved, refined and analyzed the *Leishmania *HGPRT structure. All authors contributed to writing the paper.
